# Association between *Helicobacter pylori* genotypes and severity of chronic gastritis, peptic ulcer disease and gastric mucosal interleukin-8 levels: Evidence from a study in the Middle East

**DOI:** 10.1186/s13099-014-0041-1

**Published:** 2014-09-26

**Authors:** Iqbal Siddique, Asmaa Al-Qabandi, Jaber Al-Ali, Waleed Alazmi, Anjum Memon, Abu Salim Mustafa, Thamradeen A Junaid

**Affiliations:** Department of Medicine, Faculty of Medicine, Kuwait University, P. O. Box 24923, 13110 Safat, Kuwait; Thunayan Al-Ghanim Gastroenterology Center, Al-Amiri Hospital, Sharq, Kuwait; Department of Pathology, Faculty of Medicine, Kuwait University, Jabriya, Kuwait; Division of Primary Care and Public Health, Brighton and Sussex Medical School, Brighton, UK; Department of Microbiology, Faculty of Medicine, Kuwait University, Jabriya, Kuwait

**Keywords:** *Helicobacter pylori*, *cagA*, *vacA*-s1, *vacA*-s2, Gastritis, Interleukin-8, Peptic ulcer disease

## Abstract

**Background:**

The varied clinical presentations of *Helicobacter pylori* (*H. pylori*) infection are most likely due to differences in the virulence of individual strains, which determines its ability to induce production of interleukin-8 (IL-8) in the gastric mucosa. The aim of this study was to examine association between *cagA, vacA-*s1 and *vacA-*s2 genotypes of *H. pylori* and severity of chronic gastritis and presence of peptic ulcer disease (PUD), and to correlate these with IL-8 levels in the gastric mucosa.

**Methods:**

Gastric mucosal biopsies were obtained from patients during esophagogastroduodenoscopy. The severity of chronic gastritis was documented using the updated Sydney system. *H. pylori cagA* and *vacA* genotypes were detected by PCR. The IL-8 levels in the gastric mucosa were measured by ELISA.

**Results:**

*H. pylori cagA* and/or *vacA* genotypes were detected in 99 patients (mean age 38.4±12.9; 72 males), of whom 52.5% were positive for *cagA*, 44.4% for *vacA*-s1 and 39.4% for *vacA*-s2; and 70.7% patients had PUD. The severity of inflammation in gastric mucosa was increased with *vacA*-s1 (p=0.017) and decreased with *vacA*-s2 (p=0.025), while *cagA* had no association. The degree of neutrophil activity was not associated with either *cagA* or *vacA*-s1, while *vacA*-s2 was significantly associated with decreased neutrophil activity (p=0.027). PUD was significantly increased in patients with *cagA* (p=0.002) and *vacA*-s1 (p=0.031), and decreased in those with *vacA*-s2 (p=0.011). The level of IL-8 was significantly increased in patients with *cagA* (p=0.011) and *vacA*-s1 (p=0.024), and lower with *vacA*-s2 (p=0.004). Higher levels of IL-8 were also found in patients with a more severe chronic inflammation (p=0.001), neutrophil activity (p=0.007) and those with PUD (p=0.001).

**Conclusions:**

Presence of *vacA*-s1 genotype of *H. pylori* is associated with more severe chronic inflammation and higher levels of IL-8 in the gastric mucosa, as well as higher frequency of PUD. Patients with *vacA*-s2 have less severe gastritis, lower levels of IL-8, and lower rates of PUD. The presence of *cagA* genotype is not associated with the severity of gastritis or IL-8 induction in the gastric mucosa. The association of *cagA* with PUD may be a reflection of its presence with *vacA*-s1 genotype.

## Background

*Helicobacter pylori* (*H. pylori*) colonizes the mucosa of the human stomach and establishes a long-term infection [1]. It leads to the development of chronic gastritis, peptic ulcer disease (PUD), mucosal-associated lymphoid tissue (MALT) lymphoma and gastric carcinoma [2,3]. The extent and severity of these associations depend on several elements, such as bacterial virulence factors, age of the host, genetic susceptibility, immune response and environmental factors [4–6].

The two key virulence markers of *H. pylori* are the cytotoxin associated A (*cagA*) and the vacuolating cytotoxin A (*vacA*) genes [6,7]. The *cagA* gene is not present in every *H. pylori* strain, but is associated with more severe clinical results such as more severe inflammation of the gastric mucosa, as well as higher prevalence of PUD and gastric carcinoma [8–10]. The *vacA* gene is present in all *H. pylori* strains and is associated with PUD [11]. The *vacA* gene contains at least three variable regions, the signal (s) region, intermediate (i) region and middle (m) region. The s-region exists as s1 and s2 types [12,13]. The *vacA*-s1 strains are associated with more severe gastric inflammation and PUD independently of *cagA,* while *vacA*-s2 strains are associated with lower ulcer prevalence and less severe inflammation [14].

*H. pylori* infection results in recruitment of neutrophils, lymphocytes and macrophages into the gastric mucosa through the induction of several cytokines such as TNF-α, IL-6 and IL-8 [15–17]. IL-8 is an important mediator in the immunopathogenesis of chronic gastritis caused by *H. pylori* [16]. It has been demonstrated that *cagA* and *vacA*-s1 positive strains of *H. pylori* induce production of IL-8 in the gastric mucosa, both in vivo and in vitro [16,18,19]. The *vacA*-s2 gene is not associated with IL-8 induction [18]. In addition, an association between the mucosal levels of IL-8 and severity of gastritis and presence of PUD has also been reported [19]. Most studies of association between genotypes of *H. pylori* and chronic gastritis, peptic ulcer disease and IL-8 levels have been conducted in the Western populations, and no previous study has examined these associations in the Middle East. Furthermore, the majority of published studies have only examined either a single or some of these associations.

The aim of this study was to determine the association between the presence of *cagA*, *vacA*-s1 and *vacA*-s2 genotypes in *H. pylori* and the severity of gastritis and PUD, and to correlate these with the levels of IL-8 in a group of patients from the Middle East. We have also attempted to examine all these inter-related associations in the same group of patients to validate the biologic plausibility that the bacterial virulence factors lead to induction of the cytokine IL-8, which in turn results in more severe inflammation or development of PUD.

## Results

Esophagogastroduodenoscopy and gastric biopsies were performed in 120 adult patients. *H. pylori* were seen on histopathology in 98 of these patients, all of whom were positive for *cagA* and/or *vacA*. One patient had *vacA* on PCR but was not positive for *H. pylori* on histopathology was also included in the analysis. Therefore, further analysis was carried out in these 99 patients (72.7% males, 27.3% females; mean age 38.4 years) (Table 1). A history of PUD was present in 27.3% of the patients, and the most common indication for referral was dyspepsia (84.8%).

**Table 1:** **Socio-demographic and clinical characteristics of 99 patients with** ***H. pylori*** **infection in Kuwait**

**Characteristic**			
Age at diagnosis (years)			
	Mean ± SD	38.4 ± 12.9	
	Median	35.5	
	Range	18-75	
Gender		n	(%)
	Male	72	(72.7)
	Female	27	(27.3)
Nationality			
	Kuwait	52	(52.5)
	Bangladesh	15	(15.2)
	Egypt	8	(8.1)
	State-less Arabs	5	(5.1)
	Syria	5	(5.1)
	Other^a^	14	(14.1)
Past history of peptic ulcer disease		27	(27.3)
Past history of *H. pylori* infection		4	(4.0)
History of cigarette smoking		38	(38.4)
History of alcohol consumption		6	(6.1)
Indication for esophagogastroduodenoscopy			
	Dyspepsia	84	(84.8)
	Upper gastrointestinal bleeding	6	(6.1)
	Heartburn	5	(5.1)
	Anemia	2	(2.0)
	Persistent vomiting	2	(2.0)

^a^India (4), Iran (2), Pakistan (2), Saudi Arabia (2), Afghanistan (1), Jordan (1), Somalia (1), Yemen (1).

The most frequent abnormality seen on endoscopy was PUD (70.7%) (Table 2). Endoscopic evidence of mucosal inflammation of the stomach and duodenum was observed in 57.6% and 29.3% of the patients, respectively. Chronic inflammation was “None-Mild” in 22.2% of the patients, and “Moderate-Marked” in 77.8%. Neutrophil activity was “None-Mild” in 60.6%, and “Moderate-Marked” in 39.4% of the patients.

**Table 2:** **Results of endoscopic, histological,** ***H. pylori*** **genotype, and IL-8 level in patients with** ***H. pylori*** **infection in Kuwait**

**Characteristic**				
			**n**	**%**
Esophagogastroduodenoscopy findings^a^				
	Duodenal ulcer		61	(61.6)
	Gastritis		57	(57.6)
	Duodenitis		29	(29.3)
	Gastric ulcer		13	(13.1)
	Esophagitis		10	(10.1)
	Gastric cancer		1	(1.0)
Histological findings^b^				
	*H. pylori* present		98	(99.0)
		None-Mild	44	(44.4)
		Moderate-Marked	54	(54.5)
	Chronic inflammation			
		None-Mild	22	(22.2)
		Moderate-Marked	77	(77.8)
	Neutrophil activity			
		None-Mild	60	(60.6)
		Moderate-Marked	39	(39.4)
	Glandular atrophy			
		None-Mild	65	(65.7)
		Moderate-Marked	34	(34.3)
	Intestinal metaplasia			
		None-Mild	94	(94.9)
		Moderate-Marked	5	(5.1)
*H. pylori* genotype				
	*cagA*		52	(52.5)
	*vacA*-s1		44	(44.4)
	*vacA*-s2		39	(39.4)
	*vacA*-s1 + s2		10	(10.1)
	*cagA* + *vacA*-s1		31	(31.3)
	*cagA* + *vacA*-s2		8	(8.1)
	*cagA* + *vacA*-s1 + s2		7	(7.1)
IL-8 Level(pg/mg protein)				
	Mean ± SD		1891.5 ± 1526.8	
	Median (IQR)		1567.4 (1566.3)	

^a^Some patients had more than one finding on endoscopy.

^b^According to the Updated Sydney system [40].

The presence of *cagA*, *vacA*-s1 and *vacA*-s2 genes was found in 52.5%, 44.4% and 39.4% of the patients, while 10.1% were positive for both *vacA*-s1 *vacA-*s2. The *cagA* gene was found in combination with *vacA*-s1 (*cagA* + *vacA*-s1) in 31.3% of the patients, with *vacA*-s2 (*cagA* + *vacA*-s2) in 8.1% and with both *vacA*-s1 and *vacA*-s2 (*cagA* + *vacA*-s1 + s2) in 7.1% of the patients. The IL-8 levels were available in 77 patients with a median value of 1567.4 (IQR 1566.3) pg/mg protein.

Table 3 shows the association between the *H. pylori* genotypes and severity of chronic inflammation, neutrophil activity and presence of PUD. Patients who were infected with *H. pylori* containing both the *cagA* and *vacA*-s1 genes were most likely to have “Moderate-Marked” degree of chronic inflammation (OR = 6.7, 95% CI: 1.4-31.4; p = 0.016). Patients with *vacA*-s1 gene alone also had a significantly more “Moderate-Marked” degree of chronic inflammation (OR = 3.9, 95% CI: 1.2-12.0; p = 0.017), while those with *vacA*-s2 had significantly less chronic inflammation (OR = 0.3, 95% CI: 0.1-0.8; p = 0.025). The presence of *cagA* or *vacA*-s1 genes either independently or together did not affect the severity of neutrophil activity in the gastric biopsies. Patients with *vacA*-s2 gene had a significantly less “Moderate-Marked” degree of neutrophil activity (OR = 0.3, 95% CI: 0.1-0.8; p = 0.027). The proportion of patients having PUD was highest in those with the *cagA* gene (OR = 4.8, 95% CI: 1.8-12.5; p = 0.002), followed by those with the *vacA*-s1 gene (OR = 2.8, 95% CI: 1.1-7.4; p = 0.031), while those with *vacA*-s2 had the lowest proportion of PUD (OR = 0.3, 95% CI: 0.1-0.7; p = 0.011). The presence of both *cagA* and *vacA*-s1 gene increased the risk of PUD more than the presence of these genotypes individually (OR = 6.3, 95% CI: 1.6-23.4; p = 0.006).

**Table 3:** **Association between** ***H. pylori*** **genotypes and chronic inflammation, neutrophil activity and peptic ulcer disease**

***H. pylori*** **genotype** ^**a**^ **(n)**		**Chronic inflammation** ^**b**^				**Neutrophil activity** ^**b**^				**Peptic Ulcer Disease**			
		**None-Mild**	**Moderate-Marked**	**OR (95% CI)** ^**c**^	**p-value**	**None-Mild**	**Moderate-Marked**	**OR (95% CI)** ^**c**^	**p-value**	**Absent**	**Present**	**OR (95% CI)** ^**c**^	**p-value**
		**(n 22)**	**(n 77)**			**(n 60)**	**(n 39)**			**(n 29)**	**(n 70)**		
		**n (%)**	**n (%)**			**n (%)**	**n (%)**			**n (%)**	**n (%)**		
*cagA*													
	Positive (52)	8 (15.4)	44 (84.6)	2.4 (0.9-6.7)	0.074	29 (55.8)	23 (44.2)	1.5 (0.6-3.4)	0.325	8 (15.4)	44 (84.6)	4.8 (1.8-12.5)	0.002*
	Negative (47)	14 (29.8)	33 (70.2)	Reference		31 (66.0)	16 (34.0)	Reference		21 (44.7)	26 (55.3)	Reference	
*vacA*-s1													
	Positive (44)	5 (11.4)	39 (88.6)	3.9 (1.2-12.0)	0.017*	22 (50.0)	22 (50.0)	2.2 (0.9-5.2)	0.057	8 (18.2)	36 (81.8)	2.8 (1.1-7.4)	0.031*
	Negative (55)	17 (30.9)	38 (69.1)	Reference		38 (69.1)	17 (30.9)	Reference		21 (38.2)	34 (61.8)	Reference	
*vacA*-s2													
	Positive (39)	13 (33.3)	26 (66.7)	0.3 (0.1-0.8)	0.025*	29 (74.4)	10 (25.6)	0.3 (0.1-0.8)	0.027*	17 (43.6)	22 (56.4)	0.3 (0.1-0.7)	0.011*
	Negative (60)	9 (15.0)	51 (85.0)	Reference		31 (51.7)	29 (48.3)	Reference		12 (20.0)	48 (80.0)	Reference	
*vacA*-s1 + S2													
	Positive (10)	3 (30.0)	7 (70.0)	0.5 (0.1-2.2)	0.445	8 (80.0)	2 (20.0)	0.3 (0.0-1.7)	0.191	2 (20.0)	8 (80.0)	1.8 (0.3-9.1)	0.478
	Negative (89)	19 (21.3)	70 (78.7)	Reference		52 (58.4)	37 (41.6)	Reference		27 (30.3)	62 (69.7)	Reference	
*cagA* + *vacA*-s1													
	Positive (31)	2 (6.5)	29 (93.5)	6.7 (1.4-31.4)	0.016*	18 (58.1)	13 (41.9)	0.9 (0.9-1.0)	0.737	3 (9.7)	28 (90.3)	6.3 (1.6-23.4)	0.006*
	Negative (68)	20 (20.9)	48 (70.6)	Reference		42 (61.8)	26 (38.2)	Reference		26 (38.2)	42 (61.8)	Reference	
*cagA* + *vacA*-s2													
	Positive (8)	3 (37.5)	5 (62.5)	0.4 (0.1-2.2)	0.337	5 (62.5)	3 (37.5)	0.9 (0.2-4.3)	0.959	2 (25.0)	6 (75.0)	1.2 (0.2-6.5)	0.810
	Negative (91)	19 (20.9)	72 (79.1)	Reference		55 (60.4)	36 (39.6)	Reference		27 (29.7)	64 (70.3)	Reference	
*cagA* + *vacA*-s1 + s2													
	Positive (7)	2 (28.6)	5 (71.4)	0.9 (0.9-1.0)	0.255	5 (71.4)	2 (39.4)	0.5 (0.1-3.2)	0.537	1 (14.3)	6 (85.7)	2.7 (0.3-24.5)	0.359
	Negative (92)	20 (21.7)	72 (78.3)	Reference		55 (59.8)	37 (40.2)	Reference		28 (30.4)	64 (69.6)	Reference	

^a^All patients were positive for either *cagA*, *vacA* or both.

^b^According to the Updated Sydney system [40].

^c^Age and gender adjusted odds ratio.

*Statistically significant.

**Figure 1:**
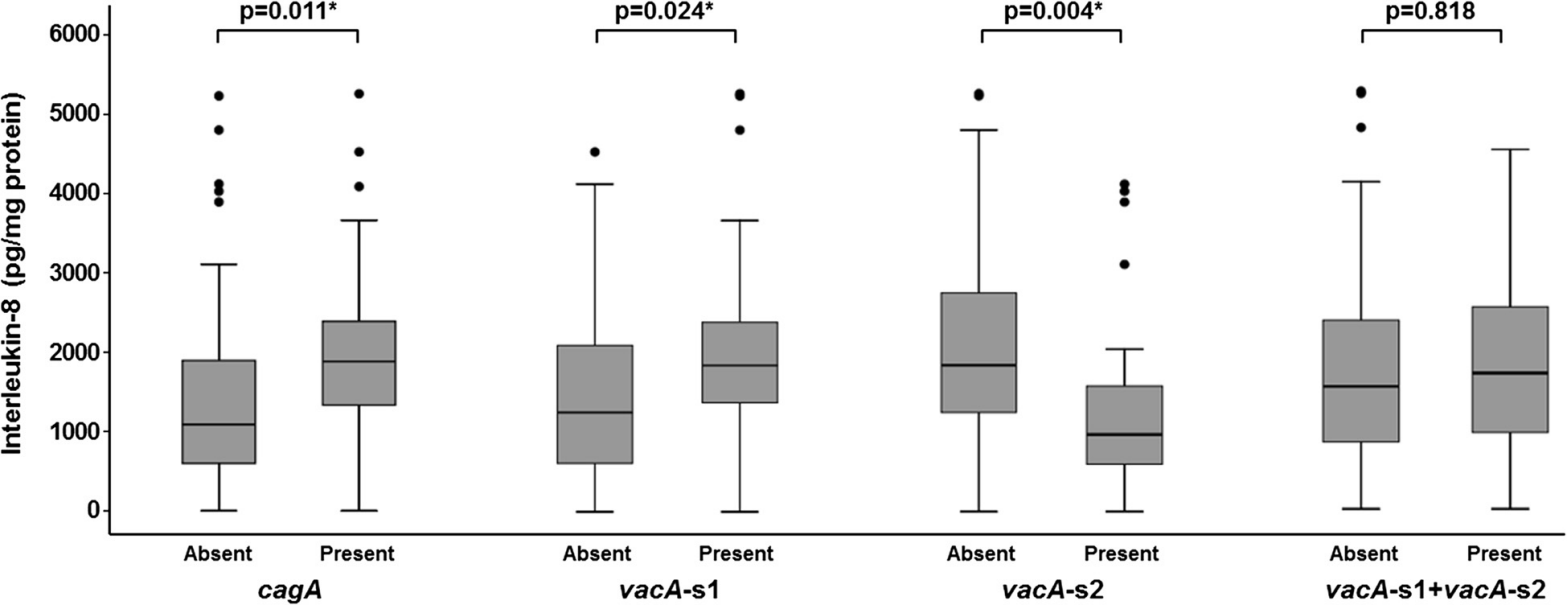
**Levels of interleukin-8 in the gastric mucosa in patients with** ***H. pylori*** **with and without genotypes** ***cagA*** **,** ***vacA*** **-s1 and** ***vacA*** **-s2.** These are expressed as box plots. The ends of the bars indicate the 25^th^ and 75^th^ percentiles. The 50^th^ percentile is indicated with a line, and the 10^th^ and 90^th^ percentiles are indicated with error bars. The p-values were calculated using the Mann–Whitney *U* test. *indicates that the p-value is statistically significant.

**Figure 2:**
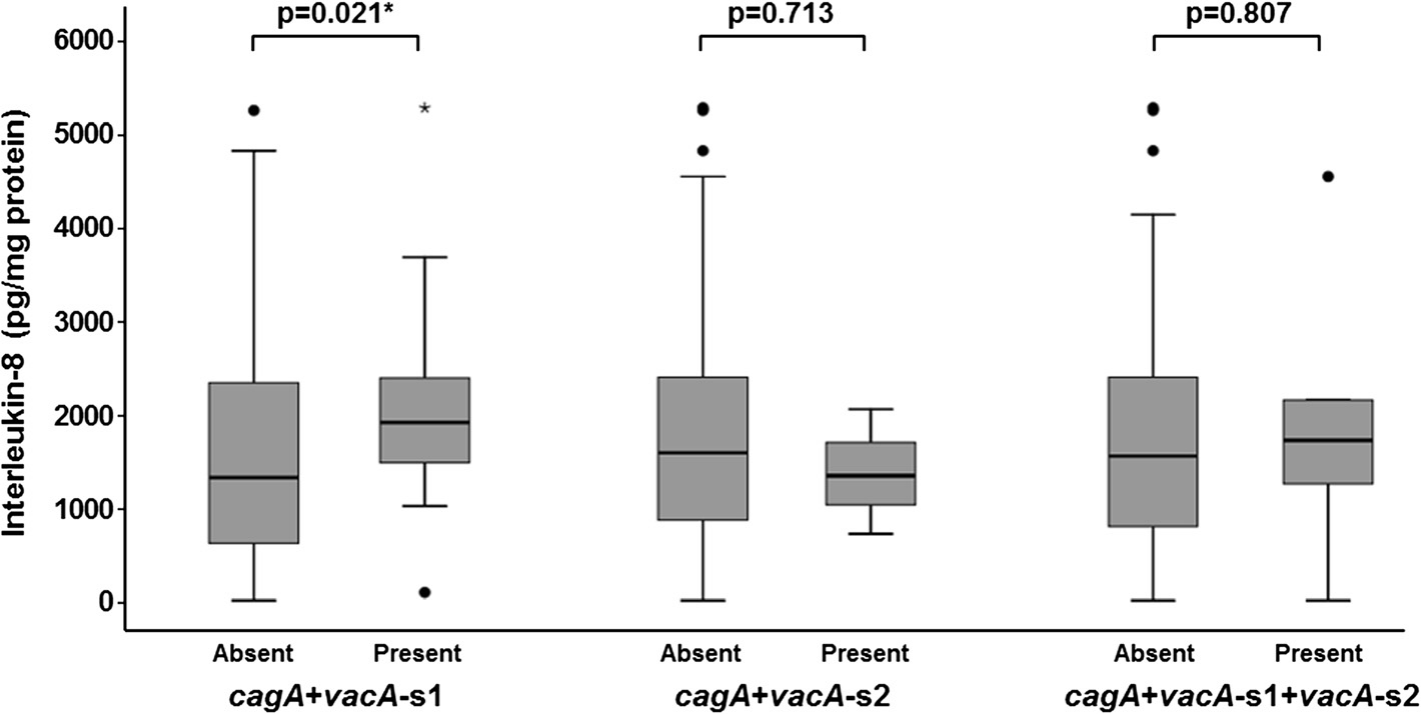
**Levels of interleukin-8 in the gastric mucosa in patients with** ***H. pylori*** **with and without genotypes** ***cagA*** **+** ***vacA*** **-s1,** ***cagA*** **+** ***vacA*** **-s2 and** ***cagA*** **+** ***vacA*** **-s1 +** ***vacA*** **-s2.** These are expressed as box plots. The ends of the bars indicate the 25^th^ and 75^th^ percentiles. The 50^th^ percentile is indicated with a line, and the 10^th^ and 90^th^ percentiles are indicated with error bars. The p-values were calculated using the Mann–Whitney *U* test. *indicates that the p-value is statistically significant.

**Figure 3:**
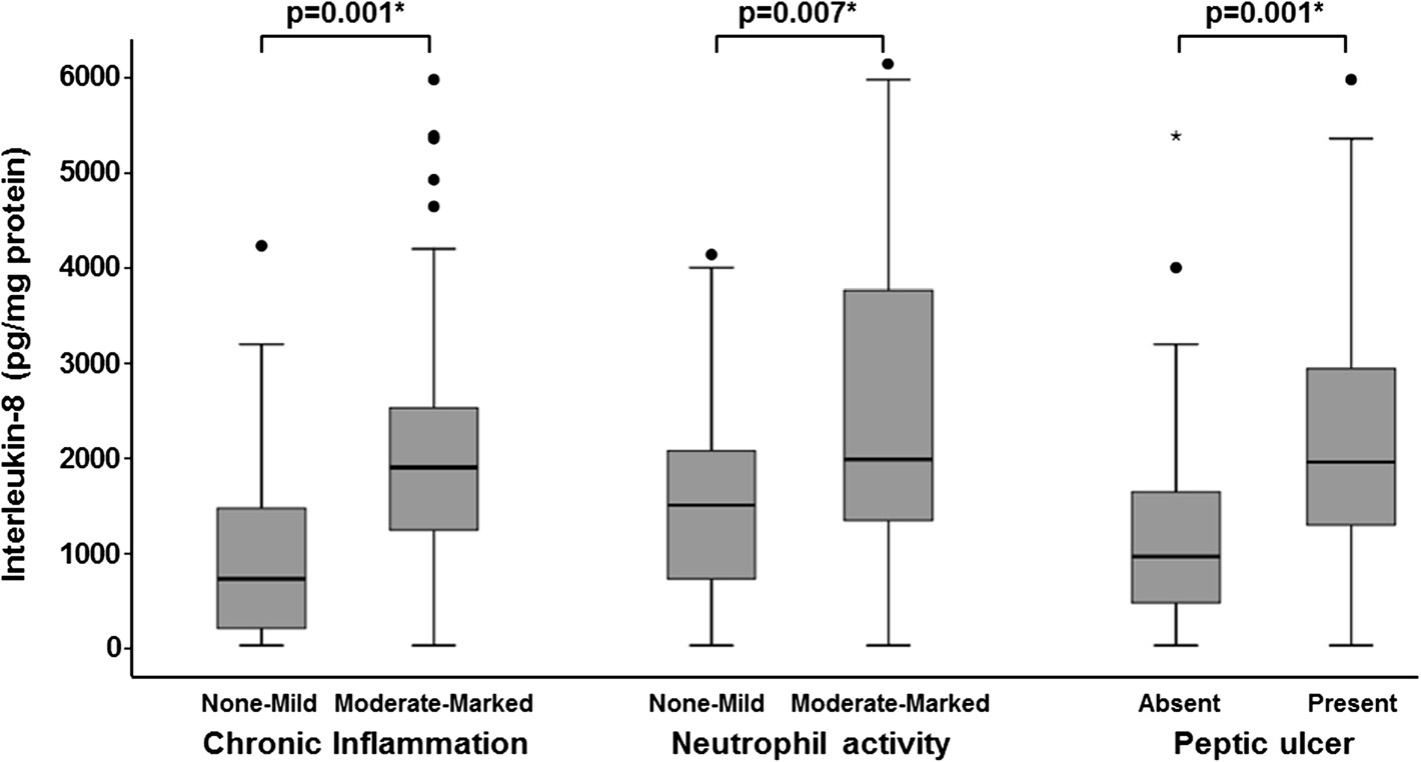
**Levels of interleukin-8 in the gastric mucosa in patients with** ***H. pylori*** **and severity of chronic inflammation, neutrophil activity and peptic ulcer disease.** These are expressed as box plots. The ends of the bars indicate the 25^th^ and 75^th^ percentiles. The 50^th^ percentile is indicated with a line, and the 10^th^ and 90^th^ percentiles are indicated with error bars. The p-values were calculated using the Mann–Whitney *U* test. * indicates that the p-value is statistically significant.

Table 4 shows the correlation between level of IL-8 in the gastric mucosa and *H. pylori* genotypes and histologic features and PUD. The median value for IL-8 was significantly higher in patients infected with *H. pylori* with *cagA* (p = 0.011) and *vacA*-s1 genes (p = 0.011 and 0.024, respectively); and significantly lower in those with *vacA*-s2 gene (p = 0.004) (Figure 1). The highest levels of IL-8 were found in patients who were positive for both *cagA* and *vacA*-s1 (Figure 2). Correlation of gastric mucosal IL-8 levels with the severity of chronic inflammation showed that patients who had “Moderate-Marked” chronic inflammation had significantly higher median IL-8 level compared to those who had “None-Mild” inflammation (p = 0.001) (Figure 3). Patients with “Moderate-Marked” neutrophil activity on gastric biopsy also had significantly higher median IL-8 levels compared to those who had “None-Mild” activity (p = 0.007). The median levels of IL-8 were also significantly elevated in the gastric biopsies of patients with PUD compared to those who did not have ulcers (p = 0.001). There was no significant difference in the density of *H. pylori* or degree of glandular atrophy or intestinal metaplasia with the IL-8 level in the gastric biopsies.

**Table 4:** **Correlation between interleukin-8 and** ***H. pylori*** **genotypes, chronic inflammation, neutrophil activity, and peptic ulcer disease**

***H. pylori*** **genotype** ^**a**^			**Interleukin-8 level** ^**b**^	**p-value** ^**c**^
			**Median (IQR)**	
	*cagA*			
		Absent	1090.3 (1310.8)	0.011*
		Present	1885.7 (1281.6)	
	*vacA*-s1			
		Absent	1245.8 (1550.2)	0.024*
		Present	1840.0 (1098.6)	
	*vacA*-s2			
		Absent	1840.0 (1597.5)	0.004*
		Present	965.6 (1205.1)	
	*vacA*-s1 + s2			
		Absent	1541.1 (1566.3)	0.818
		Present	1711.8 (1928.7)	
	*cagA* + *vacA*-s1			
		Absent	1311.8 (1814.9)	0.021*
		Present	1901.7 (915.3)	
	*cagA* + *vacA*-s2			
		Absent	1577.5 (1548.6)	0.713
		Present	1331.7 (−)	
	*cagA* + *vacA*s1 + s2			
		Absent	1541.1 (1625.9)	0.807
		Present	1711.8 (1803.6)	
Histological findings^d^				
	Chronic inflammation			
		None-Mild	689.2 (1420.6)	0.001*
		Moderate-Marked	1835.7 (1345.3)	
	Neutrophil activity			
		None-Mild	1449.0 (1342.1)	0.007*
		Moderate-Marked	1917.8 (2703.6)	
Peptic ulcer				
	Absent		919.3 (1153.3)	0.001*
	Present		1890.6 (1638.4)	

^a^All patients were positive for either *cagA*, *vacA* or both.

^b^pg/mg protein.

^c^Mann-Whitney *U* test.

^d^According to the Updated Sydney system [40].

*Statistically significant

A total of 58 (75.3%) patients who had “Moderate-Marked” chronic inflammation in the gastric mucosa had PUD, compared to 12 (54.5%) with “None-Mild”; while 31 (79.5%) patients with “Moderate-Marked” neutrophil activity had PUD, compared to 39 (65.0%) of those with “None-Mild” activity. However these differences were not statistically significant. There was also no association between the presence of PUD and severity of glandular atrophy, intestinal metaplasia or *H. pylori* density on gastric biopsies.

## Discussion

To our knowledge, this is the first study to examine the association between the presence of *cagA*, *vacA*-s1 and *vacA*-s2 genotypes in *H. pylori* and the severity of chronic gastritis and PUD in a group of patients from the Middle East. We have also demonstrated the relationship of these factors with the levels of IL-8 in the gastric mucosa validating the biologic plausibility that these bacterial virulence factors lead to induction of the cytokine IL-8, which in turn results in more severe inflammation and/or development of PUD.

There appears to be a geographic variation in the association between *H. pylori* genotypes and gastric inflammatory response. Infection with *cagA* positive strains of *H. pylori* in Western countries is reported to cause more severe gastric inflammation compared to Asian countries, where the results have been inconsistent [7,20–24]. In our study both the degree of chronic inflammation and neutrophil activity in the gastric mucosa were more severe in patients who were infected with *H. pylori* containing the *cagA* and *vacA*-s1 genes. The presence of both *cagA* and *vacA*-s1 appeared to have a synergistic role in increasing the severity of inflammation in our patients. On the other hand, the presence of *vacA*-s2 appeared to improve the severity of chronic inflammation as well as neutrophil activity regardless of the presence of *cagA*, *vacA*-s1 or both. These findings are consistent with previously published studies where *vacA*-s2 strains were found to be associated with less inflammation [25]. Both the *cagA* and *vacA*-s1 genes were also independently associated with an increased risk of PUD, while the *vacA*-s2 gene appeared to have a lesser effect. Patients who had both the *cagA* and the *vacA*-s1 gene had the highest risk of PUD. On the other hand, the presence of *vacA*-s2 decreased the risk of PUD, whether it was present alone or with *cagA*, *vacA*-s1, or both. These results are similar to those reported from the Western countries [20,21,24], even though the majority of the patients in our study were from the Middle East and the Indian Subcontinent [26]. A recent study conducted to determine the genetic affinities of *H. pylori* isolates from ethnic Arabs in Kuwait found that these strains are closely related to the Indo-European group of the organism and clearly distinct from the East Asian strains [27]. This could be the reason why the association of *cagA*, *vacA*-s1 and *vacA*-s2 and gastritis and PUD in our patients seems to be similar to that reported from the Western countries.

The pro-inflammatory cytokine IL-8 plays an important role in the regulation of mucosal neutrophil migration and activation. Our results show that the level of IL-8 in the gastric mucosa was increased in presence of *cagA* and *vacA*-s1 genotypes of *H. pylori* and the highest levels were seen in patients who were positive for both these genes. The presence of *vacA*-s2 was associated with lower levels of IL-8. Significantly higher levels of IL-8 in the gastric mucosa were also seen in patients who had more severe degree of chronic inflammation and neutrophil activity in the gastric biopsies, as well as those who had PUD. These results are in agreement with previous reports where the levels of gastric mucosal IL-8 levels have been correlated with the presence of virulence *H. pylori* genes such as *cagA* and ice A [28,29]. However, some researchers have reported no difference in the IL-8 levels in patients with *H. pylori* infections with and without PUD [30], while others have higher levels in patients with PUD then those with only gastritis [31]. This inconsistency in the results of IL-8 production could be because of the differences in the methodologies used to measure the level of the cytokine in the gastric mucosa. We have used an ELISA based technique, which may be a more sensitive method of cytokine quantification.

With reference to other factors, we found no difference in the severity of gastritis, presence of PUD or levels of IL-8 between patients with or without history of smoking or alcohol consumption. The presence of PUD was however more frequent in this group of patients than has been reported previously from Kuwait as well as elsewhere [32]. The confounding effects of medications were minimized by only selecting those patients who had not been on any of the mentioned medications for at least 4 weeks prior to endoscopy. Several other *H. pylori* factors such as *vacA*-s1 subtypes (s1a and s1b), *vacA* middle region subtypes (m1 and m2), iceA and dupA genes can also affect the virulence of this organism and its effects on the gastric mucosa [33]. However, our study was not designed to determine these virulence factors, and this could be considered a limitation of this report. Besides bacterial factors, host genetics also contribute to the pathogenesis of gastroduodenal diseases [34,35]. In addition, many cytokine gene polymorphisms reveal different risk in gastric and duodenal ulcer patients [36,37].

## Conclusions

This study which included patients from the Middle East shows that presence of *H. pylori cagA* and *vacA*-s1 genes results in induction of higher levels of the pro-inflammatory cytokine IL-8 in the gastric mucosa, which not only results in a more marked intensity of chronic inflammation and neutrophil activity in the gastric mucosa but also a higher occurrence of PUD. The presence of *vacA*-s2 results in lower levels of IL-8, as well as less severe inflammation and less PUD. In addition the presence of *vacA*-s2 appears to lessen the virulence effects of *cagA* and/or *vacA*-s1 genes.

## Patients and methods

### Patients and samples

The study population and the method of biopsy collection have been described previously [26,32]. Briefly, 120 consecutive, unselected adult patients referred for esophagogastroduodenoscopy were invited to participate in the study. Patients with coagulation abnormalities, prior gastro-duodenal surgery were excluded from the study. Patients who had taken histamine type 2 receptor antagonists, proton pump inhibitors, antibiotics, bismuth salts, aspirin or non-steroidal anti-inflammatory agents in the preceding four weeks were also excluded. The prevalence of *cagA* and *vacA* genotypes in these patients has been reported earlier [26].

Upper gastrointestinal endoscopy was performed in a standard manner. All endoscopes underwent a cleaning and disinfection process with an automated washer-disinfector before each procedure according to guidelines of the European Society of Gastrointestinal Endoscopy [38]. For each patient, four biopsies were taken from the gastric antrum, within 2 cm of the pylorus, using sterilized biopsy forceps.

Two biopsy specimens were fixed in buffered formalin, processed to paraffin, sectioned and stained with H&E and the HpSS [39]. All biopsies were examined individually by a senior gastrointestinal pathologist who was blinded to the clinical information about the patients. A biopsy was scored positive for *H. pylori* if organisms were seen on light microscopy. The updated Sydney system was used to score the density of *H. pylori* and degree of chronic inflammation, neutrophil activity, glandular atrophy and intestinal metaplasia on the biopsies [40]. Patients who had no or mild chronic inflammation in the gastric biopsy were combined in one group (None-Mild group), while those with moderate or marked degree of inflammation were included in the other group (Moderate-Marked group). Similarly, patients were divided into two groups (i.e. None-Mild and Moderate-Marked) for neutrophil activity, as well as for intestinal metaplasia and glandular atrophy.

The QIAamp® DNA mini kit (Qiagen, Hilden, Germany) was used to extract DNA from the biopsies. The purity of DNA was assessed calculating the ratio of optical density (OD) at 260–280 nm and the yield was quantified by absorbance at 260 nm.

### PCR amplification of target DNA

Table 5 shows the primers used in this study. The *cagA*F and *cagA*R primers generate a fragment of 183 base pairs (bp) for the detection of *cagA*, while primers VA1F and VA1XRA yield a 176 bp fragment for *vacA*-s1 and 203 bp for *vacA*-s2 variants [41,42]. A pre-aliquoted PCR master mix (Abgene, Surrey, UK) was used for PCR amplification of target DNA. The final reaction volume was 50 μL and contained 25pmol of each primer and 50 ng DNA [43]. PCR was performed after preincubation at 94°C for 5 minutes, followed by 35 cycles at 94°C , 50°C and 74°C for one minute each followed by a 5 minutes final extension step at 74°C. The amplified samples were analyzed by electrophoresis on 2% high-resolution agarose gel in Tris–Acetate–EDTA (TAE) buffer, which was then stained with ethidium bromide at 0.5 μg/ml [44]. Ultraviolet light was used to visualize the bands of amplified DNA, which were then photographed.

**Table 5:** **Primers used in PCR for amplification of** ***cagA*** **,** ***vacA*** **-s1 and** ***vacA*** **-s2 sequences**

**DNA region amplified**	**Primer**	**Primer sequence**	**PCR product (bp** ^**a**^ **)**
*cagA*	*cagA*F	5′-TTGACCAACAACCACAAACCGAAG-3′	183
	*cagA*R	5′-CTTCCCTTAATTGCGAGATTCC-3′	
*vacA*-s1	VA1F	5′-ATGGAAATACAACAAACACAC-3′	176
	VA1XRA	5′-CCTGAAACCGTTCCTACAGC-3′	
*vacA*-s2	VA1F	5′-ATGGAAATACAACAAACACAC-3′	203
	VA1XRA	5′-CCTGAAACCGTTCCTACAGC-3′	

^a^bp: Base pair.

### Measurement of IL-8

Two biopsy specimens were frozen immediately in liquid nitrogen and stored at −80°C. These samples were later homogenized with 1 mL phosphate buffered saline (pH 7.4) for 1 minute at 4°C. The homogenate was then centrifuged for 10 minutes at 14,000 rpm. The supernatant obtained was used for estimation of IL-8 level as well as total protein measurement [45–47]. The IL-8 concentration was measured by IL-8 ELISA kit (Immunotech, Hamburg, Germany) in a sandwich type assay using the procedure recommended by the manufacturer. The modified Lowry method was used to measure the total protein in the homogenate [48]. The amount of IL-8 in the gastric mucosal biopsies was expressed as pg/mg protein.

### Statistical analysis

The Student’s t-test was used to compare the difference between two means. We evaluated the association of PUD and severity of inflammation with the three *H. pylori* genotypes (*cagA*, *vacA*-s1 and *vacA*-s2) and their combinations. As our outcome measures were binary variables, we used univariate logistic regression models to test if any of the genotypes or their combinations are related to presence of the PUD or severity of inflammation. The strength of the association between these variables obtained from the regression models was expressed as the odds ratio (OR) and 95% Confidence Interval (95% CI) along with p-values. Further multivariate logistic regression models were used to obtain age and gender adjusted estimates of these effects. We used Mann–Whitney U tests to evaluate if the levels of IL-8 correlated with varying genotypes or their combinations, severity of inflammation and presence of PUD. We used non-parametric method as the cytokines were not normally distributed. A p-value of <0.05 was considered statistically significant. All p-values presented are two sided. The data were analyzed using the SPSS software (SPSS Inc., Chicago, IL, USA).

The protocol for the study, and the statement of informed consent, was approved by the ethical committee of the Medical Research Council of the Health Sciences Center at Kuwait University and conforms to the provisions of the World Medical Association’s Declaration of Helsinki in 1995 (as revised in Tokyo 2004). All patients gave an informed consent prior to inclusion in the study.
